# Supplementary material for: Amino Acids and Their Biological Derivatives Modulate Protein–Protein Interactions in an Additive Way
Source: J Phys Chem Lett. 2024 Jul 5;15(28):7154–60. doi: 10.1021/acs.jpclett.4c01175 (PMC11261602; doi:10.1021/acs.jpclett.4c01175)
Supplement: Supplementary file 1 — jz4c01175_si_001.pdf [file jz4c01175_si_001.pdf]

Supporting information for

"Amino Acids and Their Biological Derivatives Modulate Protein-Protein Interactions In an Additive Way"

Xufeng Xu[1*] and Francesco Stellacci[1,2*]

1: Institute of Materials, Ecole Polytechnique Fédérale de Lausanne (EPFL), Lausanne, 1015, Switzerland

2: Bioengineering Institute, Ecole Polytechnique Fédérale de Lausanne (EPFL), Lausanne, 1015, Switzerland

E-mail: xufeng.xu@ru.nl; francesco.stellacci@epfl.ch

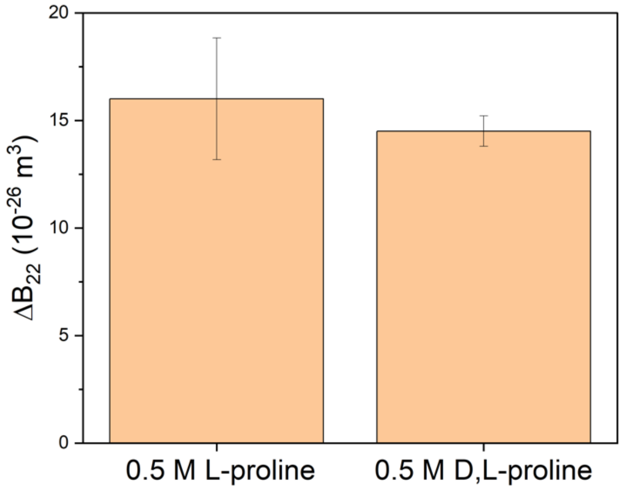

Figure S1: The effect on BSA-BSA interactions of 0.5 M L-proline and 0.5 M 1:1 racemic mixture of D- and L-proline.

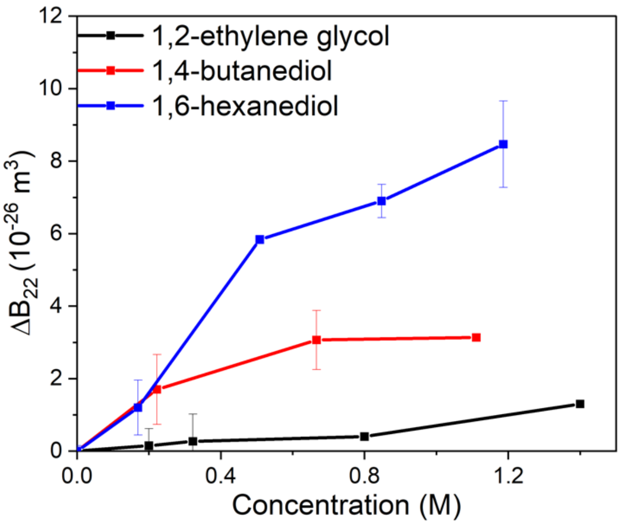

Figure S2: The effect on lysozyme-lysozyme interactions of 1,2-ethylene glycol, 1,4-butanediol, and 1,6-hexanediol.

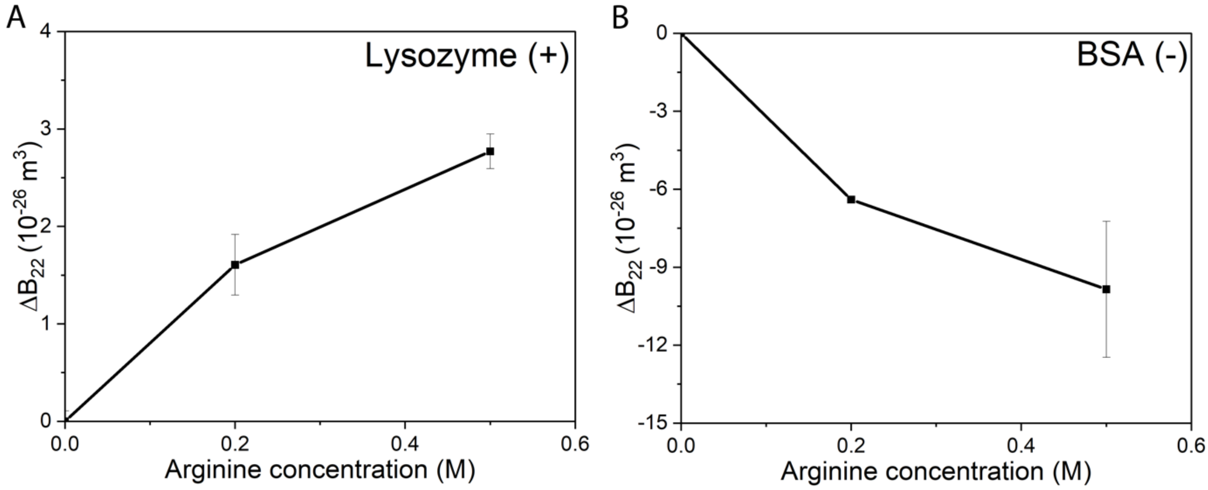

Figure S3: The effect of arginine on lysozyme-lysozyme interactions (A) and BSA-BSA interactions (B).

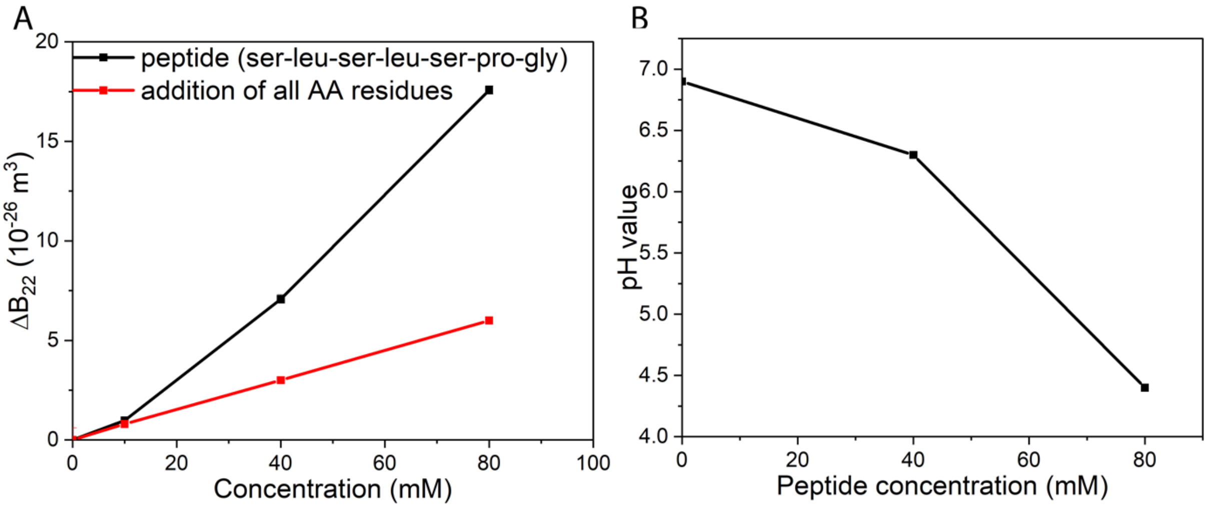

Figure S4: A. The effect on lysozyme-lysozyme interactions of heptapeptide (H-Ser-Leu-Ser-Leu-Ser-Pro-Gly-OH) compared to the sum effects of all AA residues; B. The buffer pH after the addition of the peptide.
